# Differentially expressed genes associated with high metabolic tumor volume served as diagnostic markers and potential therapeutic targets for pancreatic cancer

**DOI:** 10.1186/s12967-024-05181-z

**Published:** 2024-05-13

**Authors:** Baek Gil Kim, Sung Hwan Lee, Yeonsue Jang, Suki Kang, Chang Moo Kang, Nam Hoon Cho

**Affiliations:** 1 Brain Korea 21 PLUS Project for Medical Science, Yonsei University College of Medicine, Seoul, South Korea; 2 Department of Pathology, Yonsei University College of Medicine, Seoul, South Korea; 3 Department of Hepatobiliary and Pancreatic Surgery, Yonsei University College of Medicine, Seoul, South Korea; 4 Pancreatobiliary Cancer Center, Yonsei Cancer Center, Severance Hospital, Seoul, South Korea; 5 Division of Hepatobiliary and Pancreas, Department of Surgery, CHA Bundang Medical Center, CHA University, Pocheon, South Korea

**Keywords:** RNA sequencing, Metabolic tumor volume, Differentially expressed gene, Pancreatic cancer

## Abstract

**Background:**

The lack of distinct biomarkers for pancreatic cancer is a major cause of early-stage detection difficulty. The pancreatic cancer patient group with high metabolic tumor volume (MTV), one of the values measured from positron emission tomography—a confirmatory method and standard care for pancreatic cancer, showed a poorer prognosis than those with low MTV. Therefore, MTV-associated differentially expressed genes (DEGs) may be candidates for distinctive markers for pancreatic cancer. This study aimed to evaluate the possibility of MTV-related DEGs as markers or therapeutic targets for pancreatic cancer.

**Methods:**

Tumor tissues and their normal counterparts were obtained from patients undergoing preoperative 18F-FDG PET/CT. The tissues were classified into MTV-low and MTV-high groups (7 for each) based on the MTV2.5 value of 4.5 (MTV-low: MTV2.5 < 4.5, MTV-high: MTV2.5 ≥ 4.5). Gene expression fold change was first calculated in cancer tissue compared to its normal counter and then compared between low and high MTV groups to obtain significant DEGs. To assess the suitability of the DEGs for clinical application, the correlation of the DEGs with tumor grades and clinical outcomes was analyzed in TCGA-PAAD, a large dataset without MTV information.

**Results:**

Total RNA-sequencing (MTV RNA-Seq) revealed that 44 genes were upregulated and 56 were downregulated in the high MTV group. We selected the 29 genes matching MTV RNA-seq patterns in the TCGA-PAAD dataset, a large clinical dataset without MTV information, as MTV-associated genes (MAGs). In the analysis with the TCGA dataset, MAGs were significantly associated with patient survival, treatment outcomes, TCGA-PAAD-suggested markers, and CEACAM family proteins. Some MAGs showed an inverse correlation with miRNAs and were confirmed to be differentially expressed between normal and cancerous pancreatic tissues. Overexpression of KIF11 and RCC1 and underexpression of ADCY1 and SDK1 were detected in ~ 60% of grade 2 pancreatic cancer patients and associated with ~ 60% mortality in stages I and II.

**Conclusions:**

MAGs may serve as diagnostic markers and miRNA therapeutic targets for pancreatic cancer. Among the MAGs, KIF11, RCC1, ADCY, and SDK1 may be early diagnostic markers.

**Supplementary Information:**

The online version contains supplementary material available at 10.1186/s12967-024-05181-z.

## Background

Pancreatic cancer ranks as the seventh leading cause of cancer-related death globally [1], while it is the second to fifth leading cause in developed countries [2]. The survival rate for pancreatic cancer patients is alarmingly low, with less than 5% estimated to survive beyond 5 years [3] due to early metastasis [4, 5] and recurring instances post-surgery [6]. Diagnosis of pancreatic cancer typically occurs during advanced stages, primarily due to inconspicuous symptoms in the early phases [7], a lack of specific tumor markers [8], and challenges associated with early detection [9]. Surgical resection is currently the only treatment for pancreatic cancer, given its resistance to most current treatment options [10–12]. However, at the time of diagnosis, less than 35% of pancreatic cancer patients are eligible candidates for resection [13].

Pancreatic cancer is typically diagnosed using one or two medical imaging techniques and blood tests [14, 15]. However, these diagnostic methods have limitations and do not yield definitive results [16, 17]. Consequently, there is a growing demand for specific biomarkers, particularly for early detection purposes. While existing tumor markers have clinical utility, they are ineffective for the early diagnosis of pancreatic cancer [18]. The widely used biomarker carbohydrate antigen 19-9 (CA19-9) lacks sufficient sensitivity or specificity to aid in the early detection of pancreatic cancer. In a previous study involving 70,940 asymptomatic patients screened with a standard clinical cutoff of 37 U/ml for CA19-9, only 4 out of 1,063 patients with elevated CA19-9 (98.5% specificity) were diagnosed with pancreatic cancer, while 11 had other malignancies [19]. Another previous study screened 10,162 asymptomatic patients over the age of 40, and only four were found to have pancreatic cancer [20]. The limitations of existing tumor markers are evident, tumor marker levels can be elevated in individuals with non-cancerous diseases or without cancer, and these levels may not rise until cancer worsens or recurs after treatment [21]. Therefore, it is imperative to identify more reliable tumor markers that surpass the capabilities of conventional ones.

Positron emission tomography (PET) utilizing 2-[18F] Fluoro-2-deoxy-d-glucose (FDG) is the prevailing metabolic imaging technique widely employed in standard care for pancreatic cancer. This technology offers valuable clinical insights into the metabolic activity of cancer, facilitating differential diagnosis, preoperative staging, treatment response assessment, and prognostic prediction [22–29]. Presently, quantitative parameters employed to assess metabolic activity include the maximum standardized uptake value (SUVmax), metabolic tumor volume (MTV), and total lesion glycolysis (TLG). SUV represents the ratio of radioactivity concentration in the image to the systemic concentration of injected radioactivity. However, SUVmax alone fails to capture the complete metabolic burden of the tumor, as it only considers values from a single voxel. Moreover, SUVmax is influenced by image noise, patient characteristics, and imaging parameters [30]. MTV quantifies the volume of metabolically active tumors, while TLG is calculated as the product of mean SUV and MTV. MTV and TLG are superior to SUVmax in reflecting the metabolic tumor burden [31–33]. In a previous study, we found significant associations between MTV and TLG with radiologic tumor size and tumor differentiation, whereas SUVmax did not show such correlations. MTV demonstrated a connection with the lymph node ratio, unlike SUVmax and TLG. Furthermore, MTV emerged as a significant prognostic factor in surgically resected pancreatic cancer [34]. Given these previous findings, MTV appears strongly linked to diagnosis and prognosis. Therefore, identifying differentially expressed genes (DEGs) between the MTV-low and MTV-high groups can serve as sensitive and specific markers for pancreatic cancer.

In this study, we performed the transcriptome-wide analysis of pancreatic cancer patient tissues categorized as MTV-low and MTV-high. Subsequently, we identified the DEGs that can serve as potential markers and therapeutic targets. To assess the suitability of these DEGs, we evaluated their applicability using a large dataset that included clinical information but did not have MTV information.

## Methods

### Tissue preparation

Tumor tissues and their normal counterparts were obtained from 14 patients undergoing preoperative ^18^F-FDG PET/CT and potentially curative resection of pancreatic ductal adenocarcinoma at Severance Hospital of the Yonsei University Health System, Korea. The protocol for the study was approved by the Severance Hospital Ethics Committee (IRB number: 4-2017-0657) with a waiver of informed consent. ^18^F-FDG PET/CT imaging was performed as previously described [34]. Briefly, ^18^F-FDG PET/CT imaging was carried out with Discovery STE (GE Healthcare, Chicago, IL, USA) or Biograph TruePoint PET/CT scanner (SIEMENS Healthineers, Erlangen, Germany). The patients fasted for at least 6 h and were intravenously injected with ~ 5.5 MBq/kg of ^18^F-FDG for 60 min before imaging. CT scanning was performed at 30 mA and 130 kVp for the Discovery STE and 36 mA and 120 kVp for Biograph TruePoint without contrast enhancement. After the CT scanning was completed, PET scanning was extended from the neck to the proximal thighs for 3 min per bed in 3D mode. The images acquired from PET scanning were reconstructed with an attenuation correction using ordered subset expectation maximization (OSEM) [35]. The PET/CT images were reviewed by two board-certified nuclear medicine physicians with Advantage Workstation 4.4 (GE Healthcare, Chicago, IL, USA). Maximum standardized uptake value (SUVmax) and MTV2.5 were measured on PET images using Volume Viewer (GE Healthcare, Chicago, IL, USA). The volume of interest (VOI), including the entire lesion in the axial, sagittal, and coronal planes, was examined with a spherical shape. ^18^F-FDG uptake of normal organs such as bowel, stomach, and liver was excluded from the VOI. The patient groups with MTV-low and MTV-high were classified based on the MTV2.5 value of 4.5 (MTV-low: MTV2.5 < 4.5, MTV-high: MTV2.5 ≥ 4.5) [34].

### Total RNA sequencing

Total RNAs were extracted from the tissue samples using TRizol™ Reagent (Invitrogen, Carlsbad, CA, USA), followed by rRNA removal using Lexogen RIBO COP rRNA depletion kit (Lexogen, Vienna, Austria). The RNA quality was analyzed in NanoDrop 2000 (Thermo Fisher Scientific, Waltham, MA, USA) and Agilent 2100 Bioanalyzer (Agilent Technologies, Santa Clara, CA, USA). The RNAs above 1.8 for OD260/280, 1.6 for OD260/230, and 7.0 for RNA integrity number (RIN) were used to construct RNA libraries using SMARTer^®^ Stranded RNA-Seq Kit (Clontech Laboratories, Inc. Mountain View, CA, USA). Sequencing was performed in Hiseq 2500 System (Illumina, San Diego, CA, USA), and its base calling was carried out by Illumina Casava 1.8 software. Sequenced reads were trimmed for adaptor sequence, masked for low-complexity or low-quality sequence using the FASTX trimmer tool, and then mapped to hg19 whole genome using TopHat. Read counts were extracted and normalized using edgeR.

### Transcriptome analysis

The MTV RNA-Seq dataset was analyzed in the cancer tissue compared to its normal counterpart. To obtain significant DEGs between low and high MTV groups, we performed t-tests between the two groups and sorted the genes having below 0.05 p-value out using Excel-based Differentially Expressed Gene Analysis tool (ExDEGA, Ebiogen, Seoul, South Korea). The genes sorted out were classified into upregulated and downregulated genes in the high MTV group compared to the low MTV group and then analyzed in the Database for Annotation, Visualization and Integrated Discovery (DAVID) v2021 [36, 37]. Enrichment analysis was performed with lowest, low, medium, high, and highest stringencies. The biological processes with more than 1.3 of enrichment score, equivalent to a non-log scale of 0.05, were considered valid. The correlation of the DEGs with clinical factors was analyzed using the TCGA-PAAD, a large dataset without information about MTV. Genetic alteration analysis was performed in cBioPortal [38, 39] using the genetic alterations of the TCGA, PanCancer Atlas [40] sharing patient samples with the TCGA-PAAD. The putative miRNAs binding to the DEGs were analyzed using the miRNAs commonly predicted in miRWalk [41], TargetScan [42], and miRDB [43] databases.

### Immunohistochemical analysis

Protein expression in normal and cancer tissues was evaluated using the human protein atlas [44]. The antibodies selected for immunohistochemical analysis were as follows, HPA008572 for ITGA3, HPA004941 for KIAA1217, HPA010568 for KIF11, HPA027573 for RCC1, HPA074007 for SLC44A1, HPA023535 for SON, CAB018364 for ADCY1, HPA028803 for INPP5B, HPA011392 for SDK1.

### Graphical presentation

Cluster heatmap, bubble plot, balloon plot, Venn diagram, and enrichment bubble were drawn with the genes sorted from the transcriptome analysis using SRPLOT. Bar graph, patient survival curve, pie graph, and heatmap were visualized by GraphPad Prism 9.5.1 (GraphPad Software, La Jolla, CA, USA). The patient survival curve for the DEGs except for SNHG7 was plotted using the best expression cutoff of the Human Protein Atlas. The patient survival curve for SNHG7 was plotted using the z-score of the TCGA-PAAD. Correlation matrices were visualized using the conditional formatting function of Excel (Microsoft, Redmond, WA, USA).

### Statistical analysis

Statistical significance was determined using Student’s t-test (two-tailed, standard deviation), Pearson’s correlation coefficient, and Log-rank test. Results were considered significant at p < 0.05. All statistical analyses were performed using GraphPad Prism 9.5.1 (GraphPad Software, La Jolla, CA, USA). Asterisks were used to indicate p values: one for p ≤ 0.05, two for p ≤ 0.01, and three for p ≤ 0.001.

## Results

### The level of metabolic tumor volume was correlated with changes in gene expression and biological functions in pancreatic cancer

**Fig. 1:**
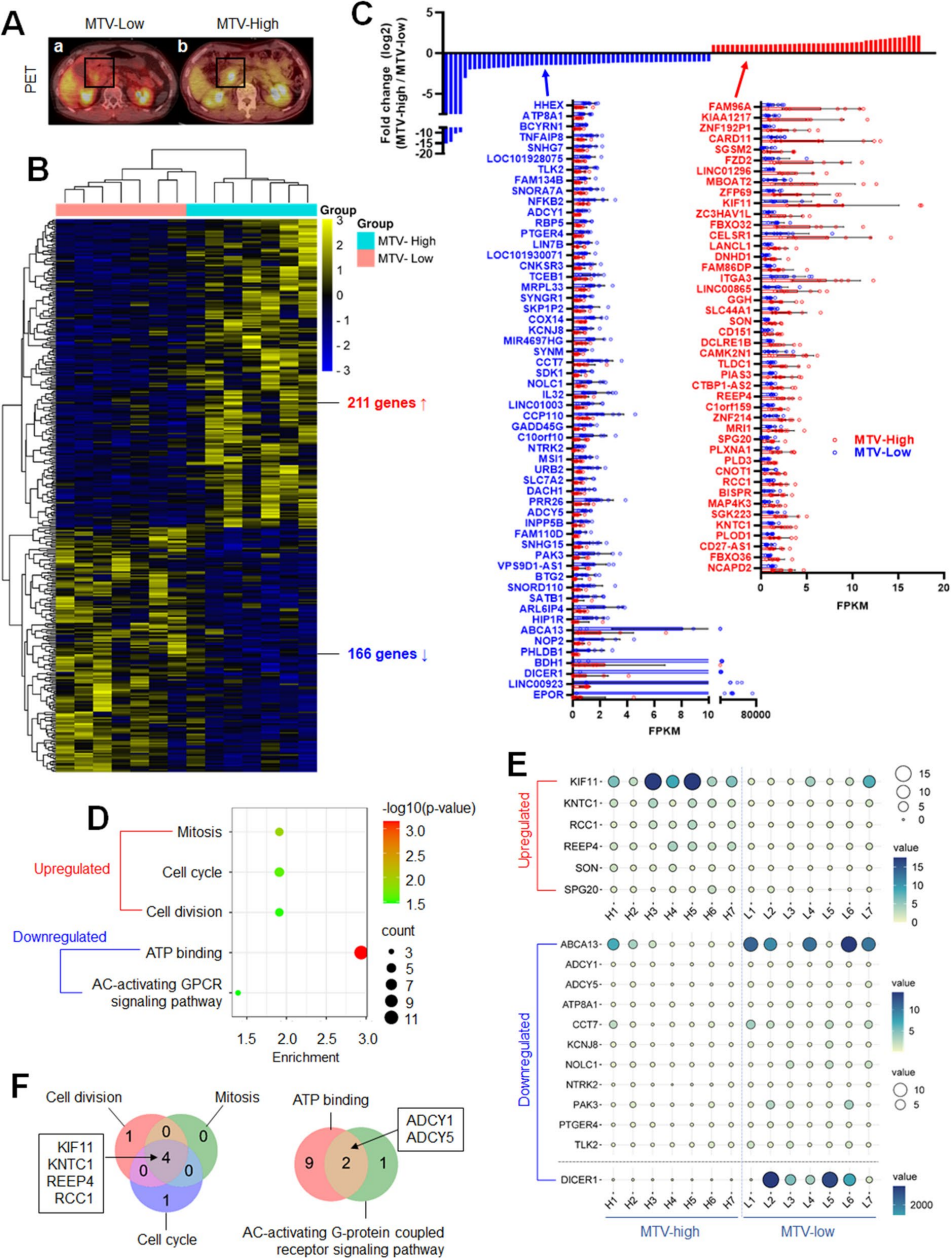
The transcriptome-wide analysis of metabolic tumor volume-associated gene expression in pancreatic cancer. **A** PET images of pancreatic cancer patients with low MTV (**a**) and high MTV (**b**). **B** Heatmap analysis of gene expression in MTV-low and -high pancreatic cancer patient tissues. 377 genes with below 0.05 p-value between MTV-low and -high groups were presented as cancer-to-normal ratio's binary logarithm. **C** Bar graphical presentation of DEG fold-change in the MTV-high group compared to the MTV-low group. The genes showing more than twofold change and a statistical significance were presented. **D** Functional annotation analysis of upregulated and downregulated genes in the MTV-high group compared to the MTV-low group. The biological processes associated with DEGs were analyzed using DAVID and presented as an enrichment bubble. **E** Balloon plot presentation of annotation-related genes in individual patients. **F** Venn diagram presentation of annotation-related genes

Pancreatic cancer patient tissues were divided into MTV-low and MTV-high groups, each containing 7 cases, based on PET images presented in Fig. 1A. We followed a five-step process outlined in Additional file 1: Fig. S1 to identify potential markers and therapeutic targets. Initially, we conducted RNA sequencing (RNA-Seq) on both normal and cancerous sections of pancreatic cancer patient tissues. This allowed us to calculate the fold change in gene expression between the cancerous and normal counterparts. The clustered heatmap revealed that, compared to the MTV-low group, the MTV-high group exhibited general upregulation of 211 genes and downregulation of 166 genes (Fig. 1B). Among the 211 upregulated genes, 44 displayed a fold difference of more than 2 and statistical significance. Similarly, among the 166 downregulated genes, 56 exhibited a fold difference of more than 2 and statistical significance. The fold change distribution was centered around twofold for both upregulated and downregulated genes, although some downregulated genes demonstrated a fold difference of 10 or more (Fig. 1C). Functional annotation analysis revealed that the upregulated genes were enriched in processes related to mitosis (KW0498), cell cycle (KW-0131), and cell division (KW-0132), while the downregulated genes were enriched in ATP binding (GO:0005524) and the adenylate cyclase (AC)-activating G-protein coupled receptor(GPCR) signaling pathway (GO:0007189) (Fig. 1D). Notably, among the genes enriched in functional annotations, Kinesin Family Member 11 (KIF11), ATP Binding Cassette Subfamily A Member 13 (ABCA13), and Dicer 1, Ribonuclease III (DICER1) exhibited very significant and dramatic differences in expression between the MTV-low and MTV-high groups (Fig. 1E). Four genes (KIF11, Kinetochore Associated 1/KNTC1, Receptor Accessory Protein 4/REEP4, and Regulator Of Chromosome Condensation 1/RCC1) were commonly associated with cell division, mitosis, and cell cycle, while two genes (Adenylate Cyclase 1/ADCY1 and Adenylate Cyclase 5/ADCY5) were involved in ATP binding and adenylate cyclase(AC)-activating G-protein coupled receptor signaling pathway (Fig. 1F).

### MTV-associated gene expression was correlated with pancreatic cancer patient survival

**Fig. 2:**
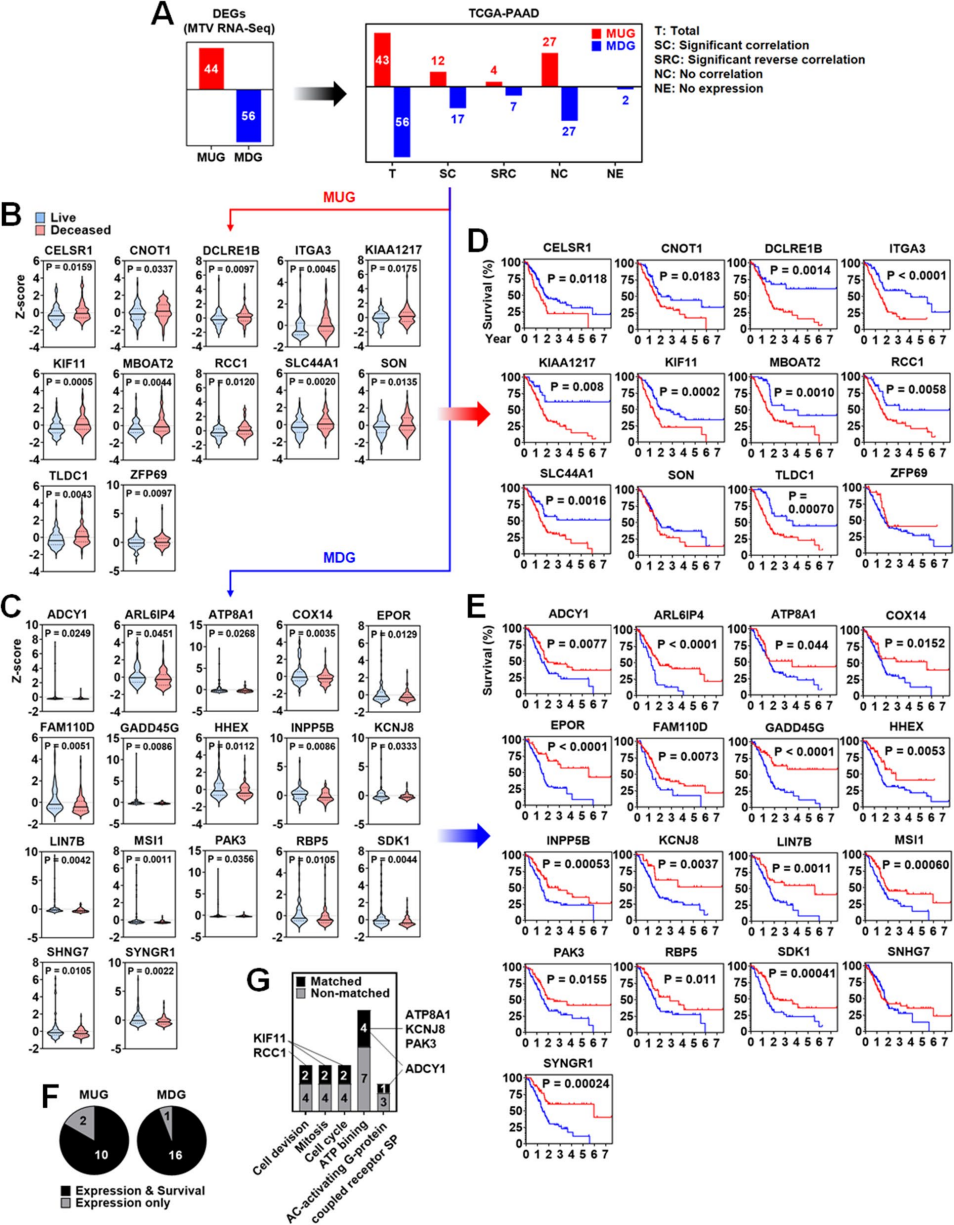
Analysis of metabolic tumor volume-associated genes in TCGA-PAAD dataset. **A** Correlation analysis between MAGs and TCGA-PAAD dataset. Expression of (**B**) MUGs and (**C**) MDGs between the live and dead groups of patients. Survival plot analysis of patient groups with low and high expression of (**D**) MUGs and (**E**) MDGs. **F** Pie graph presentation of the MUGs and MDGs showing statistically significant gene expression and patient survival. **G** Annotation-related gene analysis of TCGA-PAAD dataset

To facilitate convenience, the differentially expressed genes (DEGs) between the low and high MTV groups were designated as MTV-associated genes (MAGs), which were further divided into MTV-upregulated genes (MUGs) and MTV-downregulated genes (MDGs). To assess their potential as markers for pancreatic cancer, the expression levels of MUGs and MDGs were compared between the groups of living and deceased patients in the TCGA-PAAD dataset, a large dataset lacking MTV information. As shown in Fig. 2A, out of the 43 MUGs (excluding BST2 Interferon Stimulated Positive Regulator/BISPR, a gene not present in the dataset), 12 genes exhibited a significant positive correlation with patient death, 4 genes displayed a significant inverse correlation, and 27 genes showed no correlation. Among the MDGs, 17 genes demonstrated a significant negative correlation with patient death, 7 genes exhibited a significant inverse correlation, 27 genes showed no correlation, and 2 genes were not expressed. When comparing the living and deceased groups of pancreatic cancer patients, the deceased group displayed a significant upregulation of the MUGs (Cadherin EGF LAG Seven-Pass G-Type Receptor 1/CELSR1, CCR4-NOT Transcription Complex Subunit 1/CNOT1, DNA Cross-Link Repair 1B/DCLRE1B, Integrin Subunit Alpha 3/ITGA3, KIAA1217, KIF11, Membrane Bound *O*-Acyltransferase Domain Containing 2/MBOAT2, Regulator Of Chromosome Condensation 1/RCC1, Solute Carrier Family 44 Member 1/SLC44A1, SON DNA And RNA Binding Protein/SON, MTOR Associated Protein, Eak-7 Homolog /TLDC1/MEAK7, and ZFP69 Zinc Finger Protein /ZFP69) (Fig. 2B), while showing a significant downregulation of the MDGs (ADCY1, ADP Ribosylation Factor Like GTPase 6 Interacting Protein 4/ARL6IP4, ATPase Phospholipid Transporting 8A1/ATP8A1, Cytochrome C Oxidase Assembly Factor COX14/COX14, Erythropoietin Receptor/EPOR, Family With Sequence Similarity 110 Member D/FAM110D, Growth Arrest And DNA Damage Inducible Gamma/GADD45G, Hematopoietically Expressed Homeobox/HHEX, Inositol Polyphosphate-5-Phosphatase B/INPP5B, Potassium Inwardly Rectifying Channel Subfamily J Member 8/KCNJ8, Lin-7 Homolog B, Crumbs Cell Polarity Complex Component/LIN7B, Musashi RNA Binding Protein 1/MSI1, P21 (RAC1) Activated Kinase 3/PAK3, Retinol Binding Protein 5/RBP5, Sidekick Cell Adhesion Molecule 1/SDK1, Small Nucleolar RNA Host Gene 7/SNHG7, and Synaptogyrin 1/SYNGR1) (Fig. 2C). Additional file 2: Fig. S2 provided information on genes with no or inverse correlation. Groups with high expression of 10 MUGs exhibited significantly poorer patient survival than groups with low expression of MUGs (Fig. 2D). Similarly, groups with low expression of 16 MDGs showed significantly poorer patient survival than groups with high expression of MDGs (Fig. 2E). Furthermore, 10 MUGs and 16 MDGs displayed a significant correlation both in gene expression and patient survival (Fig. 2F). Among the 29 MAGs, 6 genes (ADCY1, ATP8A1, KCNJ8, KIF11, PAK3, and RCC1) were associated with functional annotations (Fig. 2G). Annotation-related genes other than the 6 genes did not show a significant correlation between expression and patient survival (Additional file 3: Fig. S3). In addition to differential gene expression, genetic alteration can affect the survival of patients with pancreatic cancer. Therefore, the genetic alterations of MAGs were analyzed. Of 175 patients in the TCGA-PAAD dataset, genetic alterations were found in 39 (22.3%) but not seen in 136 (77.7%) (Additional file 4: Fig. S4A). Except for MBOAT2, FAM110D, GADD45G, and ARL6IP4, amplification, deletion, and mutation (missense, splice, and truncating) occurred in MUGs and MDGs, but their frequencies were 3% or less (Additional file 4: Fig. S4B). Most of the patients with genetic alteration had less than 3 genetic alterations. However, a patient (TCGA-2J-AAB8) had 16 alterations (Additional file 4: Fig. S4C). Genetic alterations in MAGs were not associated with patient survival. There was no significant difference in patient survival between the group with alteration and non-alteration (Additional file 4: Fig. S4D).

### MTV-associated genes significantly correlated with TCGA-suggested pancreatic and blood test cancer markers

**Fig. 3:**
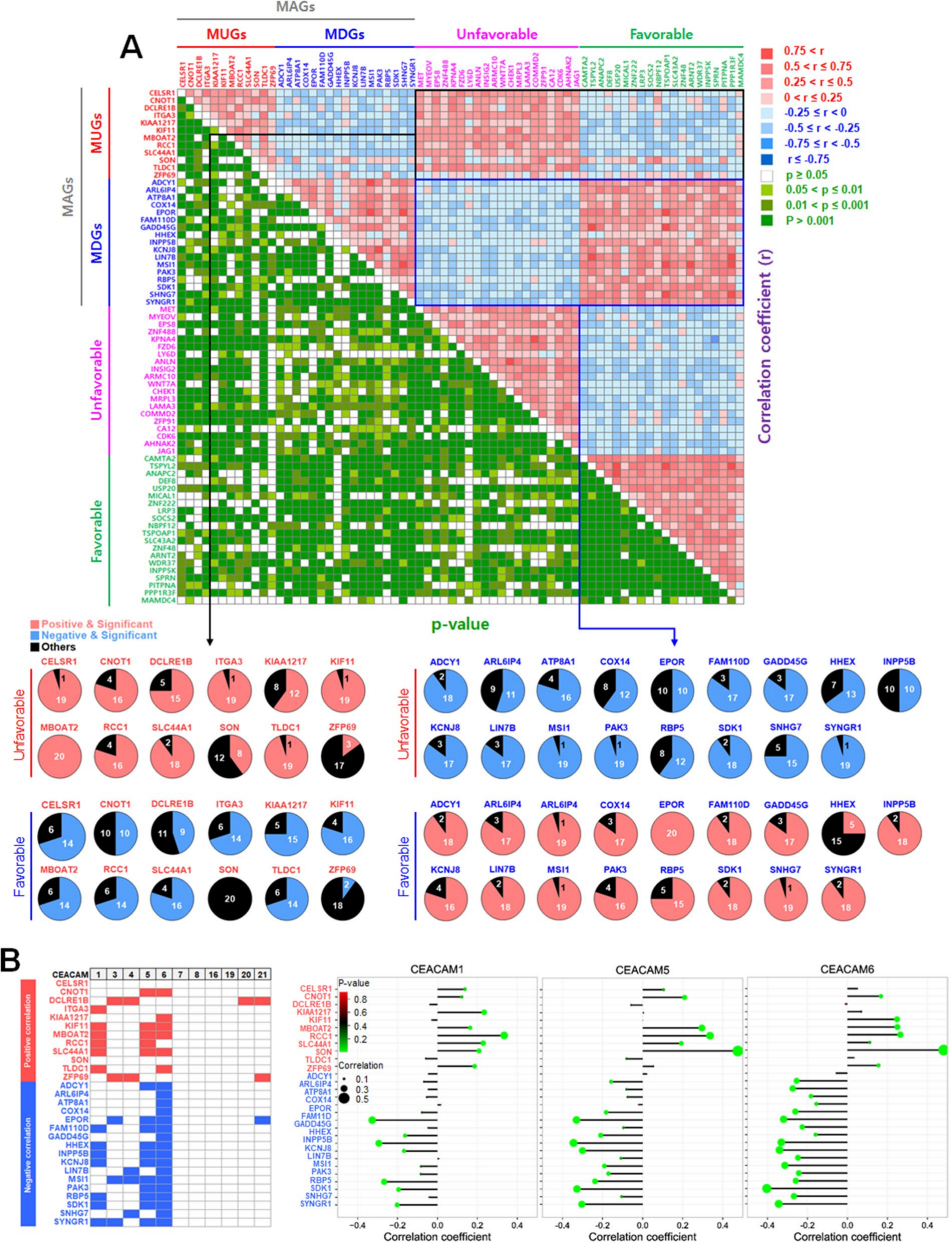
Correlation analysis between MAGs, TCGA-suggested markers, and blood test markers. **A** Correlation matrix between MAGs and the unfavorable and favorable genes of TCGA-PAAD dataset. The number of significant correlations was further presented as pie graphs. **B** Correlation analysis between MAGs and CEACAMs. CEACAM1, 5, and 6 were further presented using a correlation dot

To assess the potential of MAGs as markers for pancreatic cancer, we examined their correlation with TCGA-suggested markers, the top 20 genes associated with unfavorable and favorable prognoses based on TCGA-PAAD data analysis (The human protein atlas) [45]. As shown in Fig. 3A, most MUGs exhibited a positive correlation with unfavorable genes and a negative correlation with favorable genes. Conversely, most MDGs showed a negative correlation with unfavorable genes and a positive correlation with favorable genes. Additionally, we investigated the correlation between MAGs and CEA Cell Adhesion Molecule (CEACAMs), which are blood test markers for pancreatic cancer [46, 47]. Except for CELSR1 and SON, most MAGs correlated with CEACAM1, CEACAM3, CEACAM4, CEACAM5, CEACAM6, CEACAM20, or CEACAM21. CEACAM1, 5, and 6 notably exhibited a significant correlation with many MAGs (Fig. 3B, Additional file 5: Fig. S5A, B). Combinations of tumor markers can potentially improve cancer diagnosis and prognosis prediction compared to individual markers [48]. Therefore, we performed a correlation matrix analysis using MAGs from the MTV RNA-Seq and TCGA-PAAD datasets (deceased patient group) to identify promising combinations. In the MTV RNA-Seq, there was a clear positive correlation between MUGs or MDGs and a negative correlation between MUGs and MDGs. Similarly, the general correlation pattern in the TCGA-PAAD dataset resembled that of the MTV RNA-Seq, although there were some variations in individual correlations (Additional file 6: Fig. S6A). Significant correlations observed in the MTV RNA-Seq, TCGA-PAAD, or between the MTV RNA-Seq and TCGA-PAAD datasets were arranged as a half correlation matrix and further categorized into MTV RNA-Seq only, TCGA-PAAD only, and MTV ∩ PAAD (Additional file 6: Fig. S6B).

### Higher tumor grade and unfavorable clinical outcomes correlated with MTV-associated gene expression

**Fig. 4:**
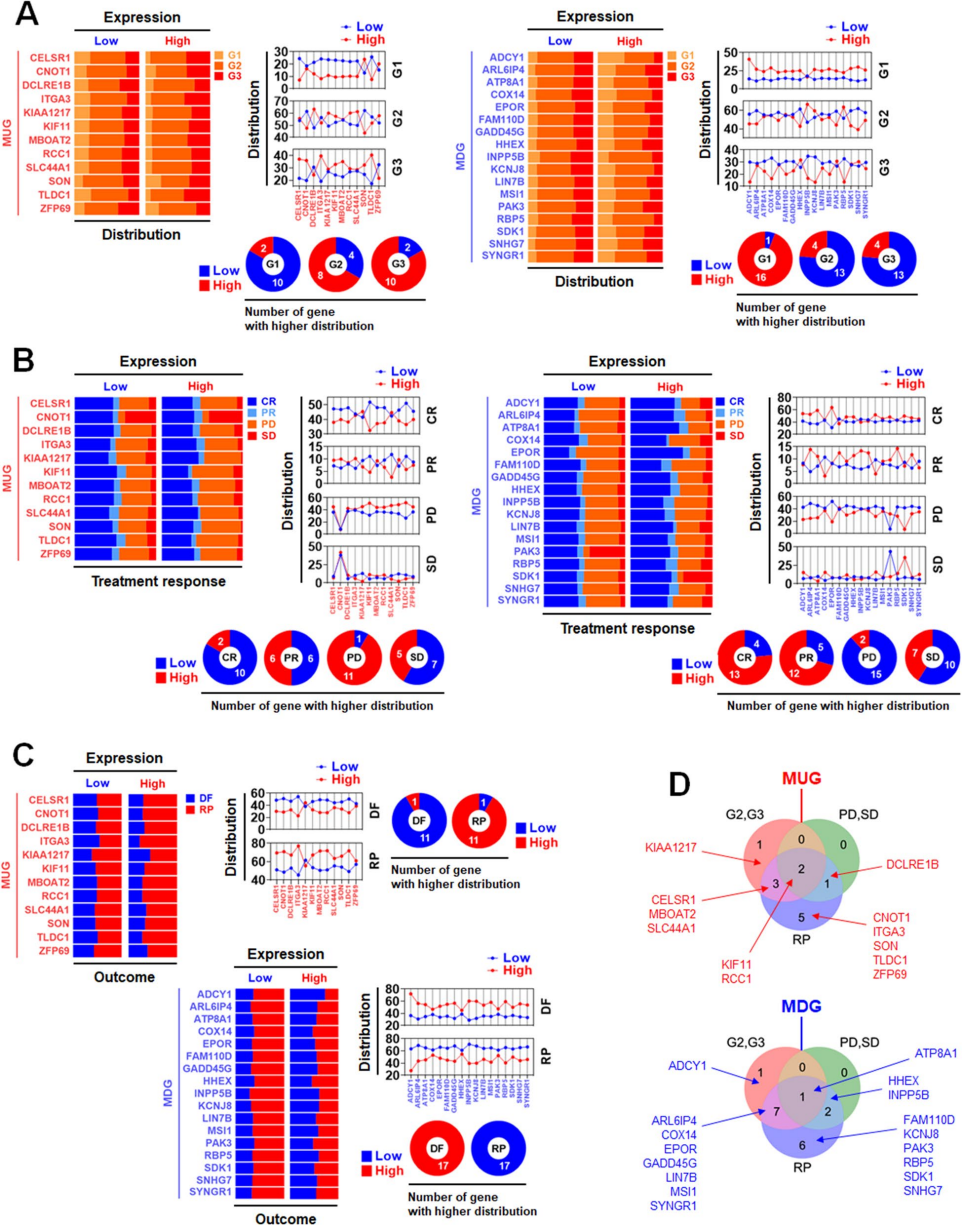
Correlation analysis of MAGs with tumor grades and clinical outcomes. Correlation of MAGs with (**A**) tumor grades, (**B**) treatment response, and (**C**) post-treatment outcomes. The correlation of MAG expression with tumor grades and clinical outcomes was presented as patient distribution (stacked bar) and distribution comparison between MAG-low and MAG-high groups (symbols with connecting lines). The number of MAGs showing a higher patient distribution for tumor grades, treatment response, and post-treatment outcomes was displayed as a pie graph. **D** Venn diagram presentation of the MAGs related to clinical outcomes

**Fig. 5:**
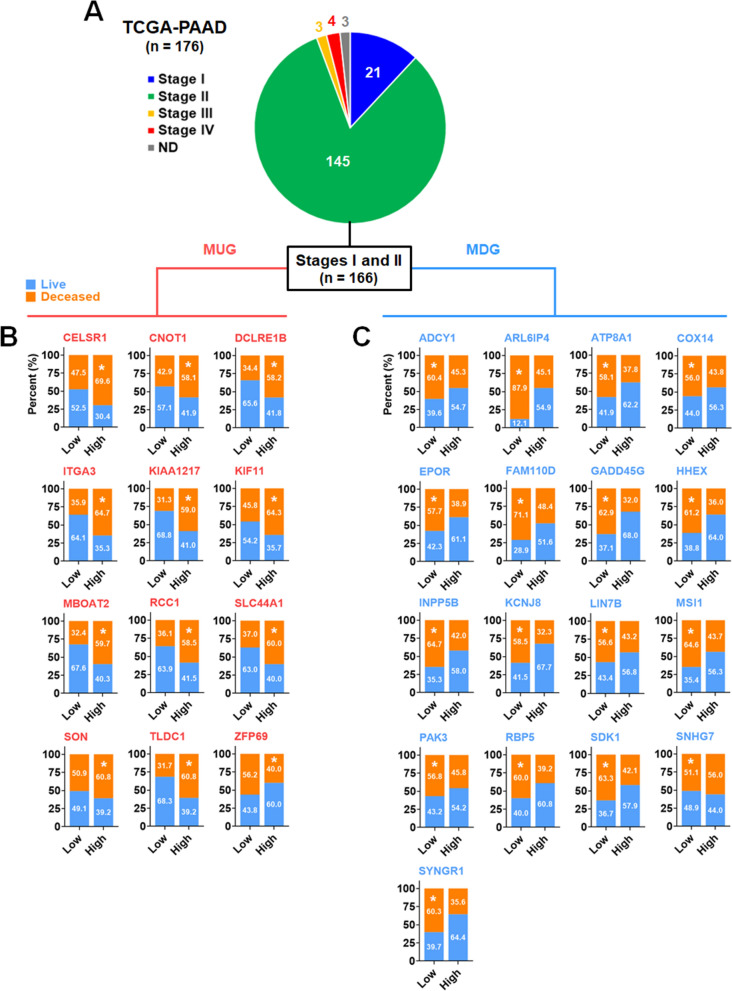
MAG expression-dependent mortality rates of stages I and II patients in the TCGA-PAAD dataset. **A** Distribution of tumor stages in the TCGA-PAAD dataset. Mortality rates of (**B**) the group with high expression of MUGs and (**C**) the group with low expression of MDGs in stages I and II pancreatic cancer patients. 166 patients in stages I or II were classified into the groups with low and high MAGs using the Human Protein Atlas cut-off values. The mortality percentages of patient groups with high MUG expression or low MDG expression were marked with a white asterisk

The presence of MAGs was observed to be correlated with patient death, as shown in Fig. 2. Therefore, we assumed that the expression of MAGs could potentially be linked to both tumor grade and clinical outcomes. In Fig. 4A, the patient group exhibiting high expression of MUGs demonstrated a greater prevalence of tumor grade (G) 2 and 3 than the group with low expression. Conversely, the patient group with low expression of MDGs exhibited a higher distribution of tumor grade 2 and 3 than the group with high expression. Tumors with higher grades displayed more upregulated MUGs and downregulated MDGs than those with lower grades. The overexpression of KIF11 and RCC1 and the underexpression of ADCY1 and SDK1 were detected in ~ 60% of tumor grade 2 pancreatic cancer patients. Treatment response was analyzed by being categorized into four groups: CR (complete regression), PR (partial regression), PD (partial disease), and SD (stable disease). In Fig. 4B, the expression of MUGs was mainly associated with CR and PD. The patient group with low MUG expression exhibited a higher distribution of CR than the group with high MUG expression, while the patient group with high MUG expression showed a higher distribution of PD than the group with low MUG expression. The expression of MDGs was also mainly associated with CR and PD. The patient group with high MDG expression displayed a higher distribution of CR than the group with low MDG expression, whereas the patient group with low MDG expression exhibited a higher distribution of PD than the group with high MDG expression. Favorable treatment responses (CR and PR) displayed more downregulated MUGs and upregulated MDGs. In contrast, unfavorable treatment responses (PD and SD) exhibited more upregulated MUGs and downregulated MDGs. Post-treatment outcomes were analyzed in DF (disease-free) and RP (recurred or progressed). As shown in Fig. 4C, the patient group with high MUG expression showed a higher distribution of RP and a lower distribution of DF, whereas the group with low MDG expression showed a higher distribution of RP and a lower distribution of DF. Among the MUGs, high expression of KIF11 and RCC1 was commonly correlated with high tumor grades (G2 and G3) and unfavorable clinical outcomes (treatment response-PD, SD, and post-treatment outcome-RP). Conversely, among the MDGs, low expression of ATP8A1 was commonly associated with tumor grades (G2 and G3) and unfavorable clinical outcomes (treatment response-PD, SD, and post-treatment outcome-RP) (Fig. 4D). To assess whether MUGs and MDGs can be used as early detection markers for pancreatic cancer, the mortality rates of stages I and II patients from the TCGA-PAAD dataset were analyzed. In Fig. 5A, ~ 94% of patients in the dataset were in stages I and II. The patients with high MUG expression (marked with a white asterisk) showed ~ 40–60% mortality (Fig. 5B). On the other hand, those with low MDG expression (marked with a white asterisk) showed around 50 to 88 percent mortality (Fig. 5C).

### Potential therapeutic options for pancreatic cancer may include miRNAs that show a reverse correlation with the MAGs

**Fig. 6:**
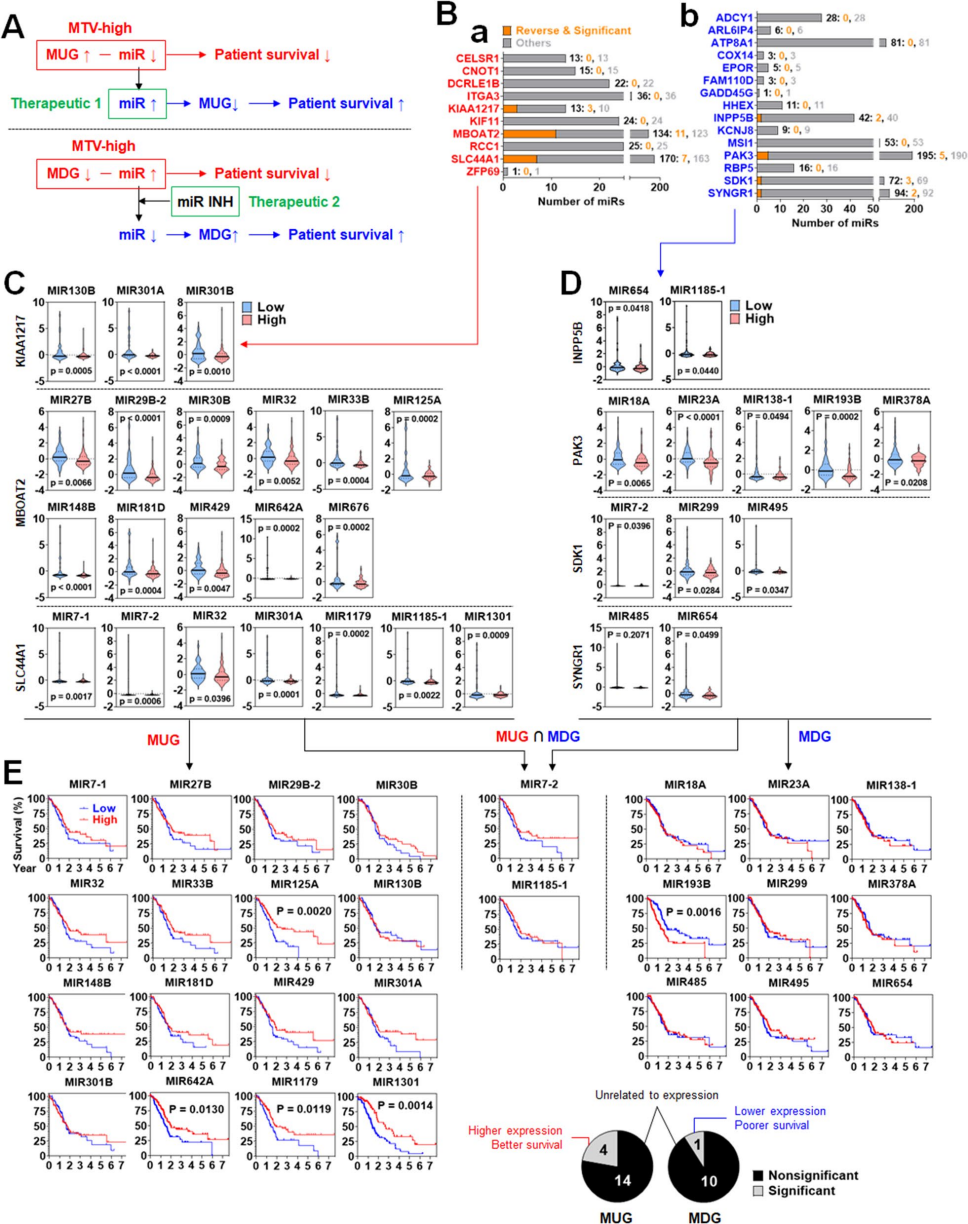
MAG-targeting miRNA expression and its association with patient survival. **A** Schematic presentation of MAG-targeting miRNA therapeutic approaches. **B** Number of the miRNAs showing reverse expression correlation with putative target MAGs. The number of total miRNAs, the number of the miRNAs showing a significant reverse expression correlation with putative target MAGs, and the number of others were labeled with black, orange, and gray, respectively. Expression of miRNAs between the patient groups with low and high expression of (**C**) MUGs and (**D**) MDGs. **E** Survival plot analysis between patient groups with low and high expression of miRNA potentially targeting MAGs. The significant and nonsignificant correlations of miRNAs with MUG or MDG were presented as pie graphs

Potential therapeutic options for pancreatic cancer could be found in the MUG-binding miRNAs or inhibitors against the MDG-binding miRNAs (Fig. 6A). Therefore, we first identified the miRNAs that potentially bind to the MAGs using a common group of three databases (miRWalk, TargetScan, and miRDB). 25 MAGs were commonly analyzed in all the databases, but 4 MAGs (LIN7B, SNHG7, SON, and TLDC1) were not (Additional file 7: Fig. S7). Subsequently, we investigated the miRNAs showing a reverse expression correlation with the 25 MAGs in the TCGA-PAAD dataset. Of the 10 MUGs, KIAA1217, MBOAT2, and SLC44A1 showed significant and inverse correlations with 3, 11, and 7 miRNAs, respectively (Fig. 6B-a). In contrast, among the 15 MDGs, INPP5B, PAK3, SDK1, and SYNGR1 exhibited significant and inverse correlations with 2, 5, 3, and 2 miRNAs, respectively (Fig. 6B-b). In Fig. 6C, the patient population with high expression of KIAA1217 demonstrated significant downregulation of miRNA(MIR)130B, MIR301A, and MIR301B compared to those with low expression. The patient population with high expression of MBOAT2 showed significant downregulation of MIR27B, MIR29B-2, MIR30B, MIR32, MIR33B, MIR125A, MIR148B, MIR181D, MIR429, MIR642A, and MIR676 compared to those with low expression. The patient population with high expression of SLC44A1 displayed significant downregulation of MIR7-1, MIR7-2, MIR32, MIR301A, MIR1179, MIR1185-1, and MIR1301 compared to those with low expression. In Fig. 6D, the patient population with low expression of INPP5B demonstrated significant upregulation of MIR654 and MIR1185-1 compared to those with high expression. The patient population with low expression of PAK3 exhibited significant upregulation of MIR18A, MIR23A, MIR138-1, MIR193B, and MIR378A compared to those with high expression. The patient population with low expression of SDK1 showed significant upregulation of MIR7-2, MIR299, and MIR495 compared to those with high expression. The patient population with low expression of SYNGR1 displayed significant upregulation of MIR485 and MIR654 compared to those with high expression. To assess the risk of the therapeutic manipulation of corresponding miRNAs, we analyzed patient survival based on the low and high expression of the miRNAs. Patient survival was classified into three groups: MUG (16 miRNAs), MUG ∩ MDG (2 miRNAs), and MDG (9 miRNAs). As shown in Fig. 6E, the patient group with high expression of miRNAs targeting the MUGs did not show a significantly poorer survival than those with low expression. The expression of miRNAs commonly involved in MUG and MDG did not significantly affect patient survival. Except for one miRNA, MIR193B, the patient group with low expression of miRNAs targeting the MDG did not show a significantly poorer survival than those with high expression. The Patients with high expression of MIR193B displayed better survival than those with low expression. Therefore, the inhibition of MIR193B may not be helpful for patient survival.

### MAG proteins may be specific markers and therapeutic targets for pancreatic cancer

**Fig. 7:**
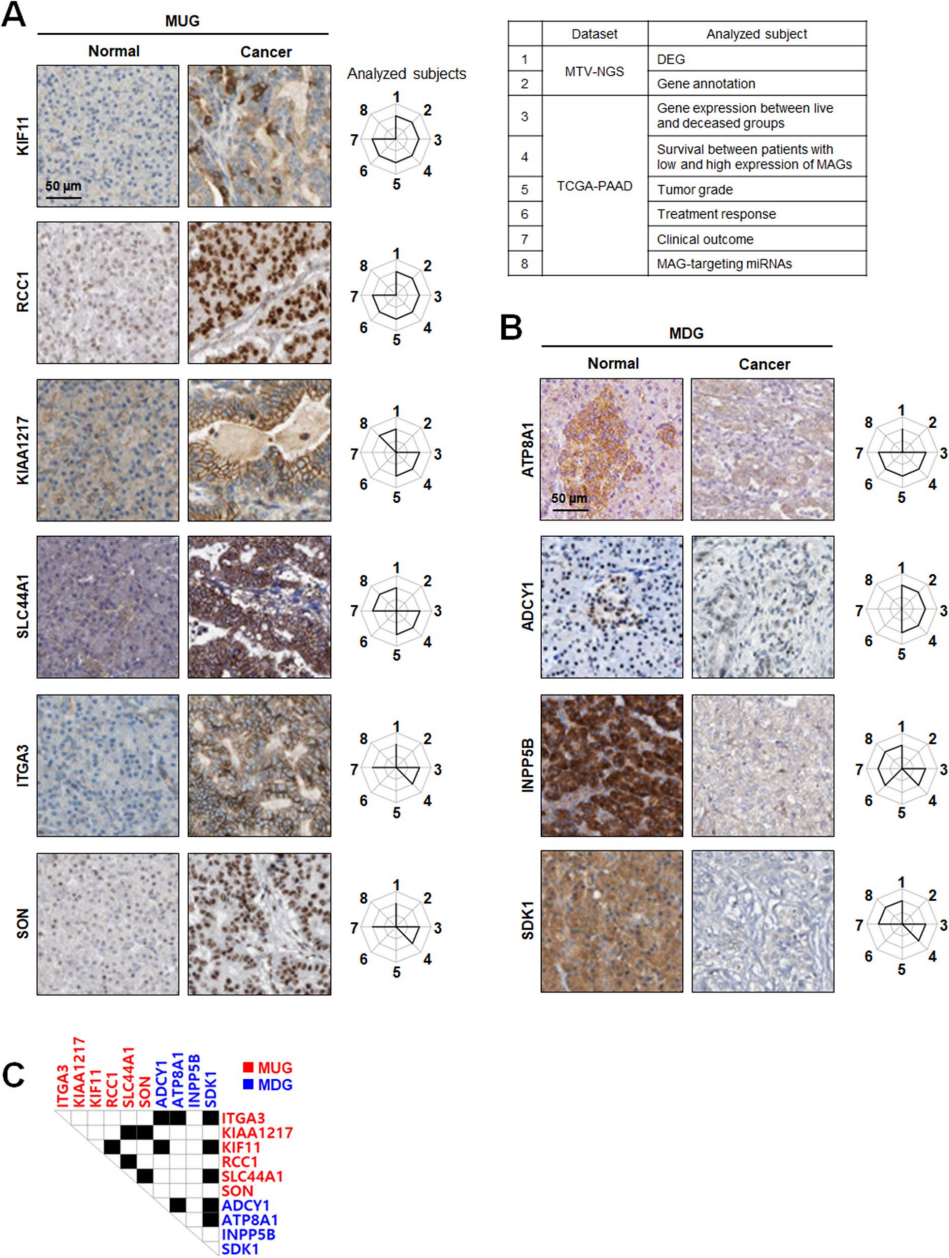
Expression analysis and combinable sets of MAG proteins. Immunohistochemical staining of (**A**) MUGs and (**B**) MDGs in pancreatic normal and cancer tissues. **C** Transcriptome analysis-based combinable sets of MAG proteins for markers and therapeutic targets

The MAGs validated in the TCGA-PAAD dataset were protein-coding genes, except for SHNG7 (long non-coding RNA). Therefore, their protein expression was analyzed between pancreatic normal and cancer tissues using the human protein atlas. In Fig. 7A, KIF11, RCC1, KIAA1217, SLC44A1, ITGA3, and SON were overexpressed in cancer tissues compared to normal ones. Among those MUG proteins, KIF11 and RCC1 were the most validated markers according to the number of analyzed subjects. In Fig. 7B, compared to normal tissues, ATP8A1, ADCY1, INPP5B, and SDK1 were underexpressed in cancer tissues. Among those MDG proteins, ATP8A1 and INPP5B were the most validated markers according to the number of analyzed subjects. Like the correlation matrix analysis with MAG transcripts, the possible combinations of MAG proteins were presented. ITGA3 was combinable with ADCY1, ATP8A1, and SDK1. KIAA1217 was combinable with SLC44A1 and SON. KIF11 were combinable with RCC1, ADCY1, and SDK1. SLC44A1 was combinable with SON and SDK1. ADCY1 was combinable with ATP8A1 and SDK1. ATP8A1 was combinable with SDK1 (Fig. 7C). To evaluate the potential of the MAG combinations in the early detection of pancreatic cancer, patient mortality rates were analyzed using stages I and stage II patients from the TCGA-PAAD dataset. In Additional file 8: Fig. S8, the MUG x MUG combination showed ~ 60–70% mortality, the MDG × MUG combination showed ~ 66–70% mortality, and the MDG × MDG combination showed ~ 63–66% mortality.

## Discussion

The functional analysis enriched the MUGs in mitosis, cell cycle, and cell division (Fig. 1D). The uncontrolled growth of cells and the instability of genetic material, which are fundamental characteristics of cancer, are linked to an increase in mitosis, cell cycle, and cell division [49–51]. Hence, the excessive expression of the MUGs could potentially contribute to the advancement of pancreatic cancer cells. On the other hand, the MDGs were found to be associated with ATP binding and AC-activating GPCR signaling pathway. ATP binding is necessary for ATP-binding cassette (ABC) transporters [52], including ABCC1, a transporter that is known to be associated with multidrug resistance [53]. Therefore, reducing the expression of the MDGs does not appear to help address drug resistance in pancreatic cancer. However, it is noteworthy that cytoplasmic ATP induces structural alterations in GLUT, inhibiting glucose uptake [54], which can potentially impede cell proliferation in cancer. Additionally, cyclic AMP (cAMP), a product of adenylyl cyclase, suppresses tumor growth in various human cancers [55]. The decrease in cAMP production may be associated with inhibiting tumor suppressor functions. Therefore, the downregulation of MDGs may play a role in the progression of pancreatic cancer.

The MAGs may be therapeutic targets for pancreatic cancer as well as markers. Since the miRNA dataset of the same patients was available, we mined the putative miRNAs binding to the MAGs that can be used as therapeutic agents or targets. For the MUGs, reversely downregulated miRNAs in the MUG-high group can be considered potential candidates for therapeutic miRNAs. KIAA1217, MBOAT2, and SLC44A1 showed a significant reverse correlation with the candidates (Fig. 6B-a). According to previous studies, KIAA1217 is a fusion partner of rearranged during transfection (RET) fusion gene, and its fusion to RET leads to oncogenic transformation in lung cancer [56]. MBOAT2, exactly Circ-MBOAT2 (Circular RNA/CircRNA bound MBOAT2), was significantly upregulated in pancreatic cancer tissues compared to normal tissues and associated with pancreatic cancer progression [57]. SLC44A1, a choline transporter-like protein, is overexpressed in various cancer cell lines [58–61]. For the MDGs, reversely upregulated miRNAs in the MDG-low group can be considered the potential targets of therapeutic inhibitors such as anti-miRNAs. INPP5B, PAK3, SDK1, and SYNGR1 showed a significant reverse correlation with the target miRNAs (Fig. 6B-b). The decreased expression of INPP5B is associated with poor prognosis in lung adenocarcinoma [62]. The loss of PAK3, one of the serine/threonine kinase family members, prevents cancer cell growth in the absence of p53 [63]. A significantly longer PFS was observed in patients with a low expression of SDK1 [64]. The expression of SYNGR1 was downregulated in bladder cancer [65]. Interestingly, the differential expression of the corresponding miRNAs was not significantly associated with patient survival, which showed that the miRNAs can be therapeutics with low side effects.

According to immunohistochemical analysis, KIF11, RCC1, KIAA1217, SLC44A1, ITGA3, SON, ATP8A1, ADCY1, INPP5B, and SDK1 were differentially expressed in protein levels between pancreatic normal and cancer tissues and also validated by multiple analyses (Fig. 7A, B). In particular, the overexpression of KIF11 and RCC1 and the underexpression of ADCY1 and SDK1 were detected in ~ 60% of tumor grade 2 pancreatic cancer patients (Fig. 4A) and associated with around 60% mortality in stages I and II (Fig. 5B, C), which shows they can be early-diagnosis markers for pancreatic cancer. MAG proteins can make some combinations to improve cancer diagnosis and prognosis prediction, and SDK1 was the most combinable partner among them (Fig. 7C and Additional file 8: Fig. S8). KIAA1217, SLC44A1, INPP5B, and SDK1 were reversely correlated with some miRNAs not affecting patient survival (Fig. 6E), which shows these are potential therapeutic targets for pancreatic cancer.

## Conclusions

Due to the lack of specific tumor markers, pancreatic cancer is generally diagnosed at an advanced stage which is impossible for surgical resection, the only currently available treatment option. For this reason, the discovery of pancreatic cancer-specific markers is a crucial issue. Metabolic tumor volume is significantly associated with diagnostic and prognostic factors, which may mean that DEGs in the groups with low or high MTV may be pancreatic cancer-specific markers. Therefore, we obtained DEGs from a comparative analysis of the RNA-Seq data acquired from pancreatic cancer patients with low or high MTV and evaluated their availability as pancreatic cancer markers on the TCGA-PAAD dataset, a large dataset including clinical information but not MTV information. There were limitations in this study, such as candidate mining from a small sample pool and lack of functional validation. Nevertheless, multiple analytic approaches with transcriptome, clinical information, and immunohistochemistry may support that our suggested marker and therapeutic candidates were reliable and valuable for further investigation. In conclusion, the MAGs can be early-diagnosis markers and potential targets of miRNA-based therapeutics for pancreatic cancer.

### Supplementary Information

**Additional file 1: Figure S1. **Data analysis workflow. Step 1. MTV RNA-Seq analysis. The initial stage involved conducting MTV RNA-Seq analysis to examine the differentially expressed genes (DEGs) in the MTV-high patient group compared to the MTV-low patient group. Subsequently, these DEGs were further studied to identify and analyze potential biological processes associated with them. Step 2. Application of MTV RNA-Seq analysis to the TCGA-PAAD dataset. The DEGs identified in Step 1 were further analyzed within the TCGA-PAAD dataset, which does not contain MTV information, to assess whether these DEGs could serve as reliable markers. The DEGs were designated as MTV-associated genes (MAGs) consisting of MTV-upregulated genes (MUGs) and MTV-downregulated genes (MDGs). Step 3. Correlation analysis between the DEGs and clinical outcomes. Using the clinical information of TCGA-PAAD, the correlation between MAGs and clinical outcomes was analyzed. Step 4. Potential miRNA therapeutics. MAG-coupled miRNAs' expression and patient survival were analyzed to find the potential miRNA therapeutics for pancreatic cancer. Step 5. Validation in protein levels. MAG expression was compared between pancreatic normal and cancer tissues and confirmed whether it matched the transcriptome analysis.

**Additional file 2: Figure S2.** Expression graphs of MAGs that did not match the MTV RNA-Seq analysis. Expression graphs of A) MUGs and B) UDGs between live and deceased groups. Red-boxed genes are included in the enriched annotation.

**Additional file 3: Figure S3.** The survival comparison of patient groups with low and high expression of the genes involved in annotation but not MAGs.

**Additional file 4: Figure S4.** The genetic alteration of MAGs and its association with patient survival. A) Venn diagram presentation of the number of patients with or without MAG-related genetic alteration in TCGA-PAAD. B) The frequency of MAG-related genetic alterations in TCGA-PAAD. C) The distribution of MAG-related genetic alterations in patients. D) Survival comparison between the patient groups with or without MAG-related genetic alteration.

**Additional file 5: Figure S5. **Correlation heatmap between MAGs and CEACAMs. The heatmap presentation of A) correlation coefficients (r) and B) p-values between MAGs and CEACAMs.

**Additional file 6: Figure S6.** Correlation analysis of MAGs between MTV RNA-Seq and TCGA-PAAD dataset. A) The correlation matrix analysis of MAGs in MTV RNA-Seq and TCGA-PAAD. B) Statistically significant correlation in MTV RNA-Seq and TCGA-PAAD. The correlation analyses were presented in four categories, (1) MTV ∪ PAAD, (2) MTV RNA-Seq only, (3) TCGA-PAAD only, and (4) MTV ∩ PAAD.

**Additional file 7: Figure S7.** Venn diagram presentation of the databases analyzed for identifying the miRNAs targeting MAGs. 25 MAGs were commonly involved in all three databases. 3 genes were included in only two databases (miRWalk and miRDB). SNHG7 was not found in all three databases.

**Additional file 8: Figure S8.** MAG combination-predicted mortality rates in stages I and II patients in the TCGA-PAAD dataset. The mortality rates were calculated in the patient group showing high expression of two MUGs for the MUG x MUG combination, low expression of an MDG and high expression of a MUG for the MDG x MUG combination, and low expression of two MDGs for the MDG x MDG combination. The numbers and colors of the half matrix match those of the bar graphs. Abbreviation: L-L: a combination of two low-expression MAGs; H-H: a combination of two high-expression MAGs; L-H: a combination of a low-expression MAG and a high-expression MAG; and H-L: a combination of a high-expression MAG and a low-expression MAG. White asterisks indicate the mortality rates calculated by MAG combinations.

## Data Availability

The raw and processed data of the MTV RNA-Seq are available in the GEO database (GSE192647).

## Abbreviations

18F-FDG PET/CT: 18F-fluorodeoxyglucose positron emission tomography/computed tomography
ABCA13: ATP Binding Cassette Subfamily A Member 13
ADCY1: Adenylate cyclase 1
ADCY5: Adenylate cyclase 5
ARL6IP4: ADP ribosylation factor like GTPase 6 interacting protein 4
ATP8A1: ATPase phospholipid transporting 8A1
BISPR: BST2 interferon stimulated positive regulator
CA19-9: Carbohydrate antigen 19-9
CEACAM: CEA cell adhesion molecule
CELSR1: Cadherin EGF LAG seven-pass G-type receptor 1
CNOT1: CCR4-NOT transcription complex subunit 1
COX14: Cytochrome C oxidase assembly factor COX14
CR: Complete regression
DCLRE1B: DNA cross-link repair 1B
DEG: Differentially expressed gene
DICER1: Dicer 1, ribonuclease III
EPOR: Erythropoietin receptor
FAM110D: Family with sequence similarity 110 member D
GADD45G: Growth arrest and DNA damage inducible gamma
HHEX: Hematopoietically expressed homeobox
INPP5B: Inositol polyphosphate-5-phosphatase B
ITGA3: Integrin subunit alpha 3
KCNJ8: Potassium inwardly rectifying channel subfamily J member 8
KIF11: Kinesin family member 11
KNTC1: Kinetochore associated 1
LIN7B: Lin-7 homolog B, crumbs cell polarity complex component
MBOAT2: Membrane bound *O*-acyltransferase domain containing 2
MIR: MiRNA
MSI1: Musashi RNA binding protein 1
MTV: Metabolic tumor volume
PAK3: P21 (RAC1) activated kinase 3
PD: Partial disease
PR: Partial regression
RBP5: Retinol binding protein 5
RCC1: Regulator of chromosome condensation 1
SD: Stable disease
SDK1: Sidekick cell adhesion molecule 1
SLC44A1: Solute carrier family 44 member 1
SNHG7: Small nucleolar RNA host gene 7
SON: SON DNA and RNA binding protein
SUVmax: Maximum standardized uptake value
SYNGR1: Synaptogyrin 1
TCGA-PAAD: The Cancer Genome Atlas Pancreatic Adenocarcinoma
TLDC1/MEAK7: MTOR associated protein, Eak-7 homolog
TLG: Total lesion glycolysis
ZFP69: ZFP69 zinc finger protein
