# Supplementary figures and images for: Differentially expressed genes associated with high metabolic tumor volume served as diagnostic markers and potential therapeutic targets for pancreatic cancer
Source: J Transl Med. 2024 May 13;22:453. doi: 10.1186/s12967-024-05181-z (PMC11092202; doi:10.1186/s12967-024-05181-z)

Supplementary Figure S1.

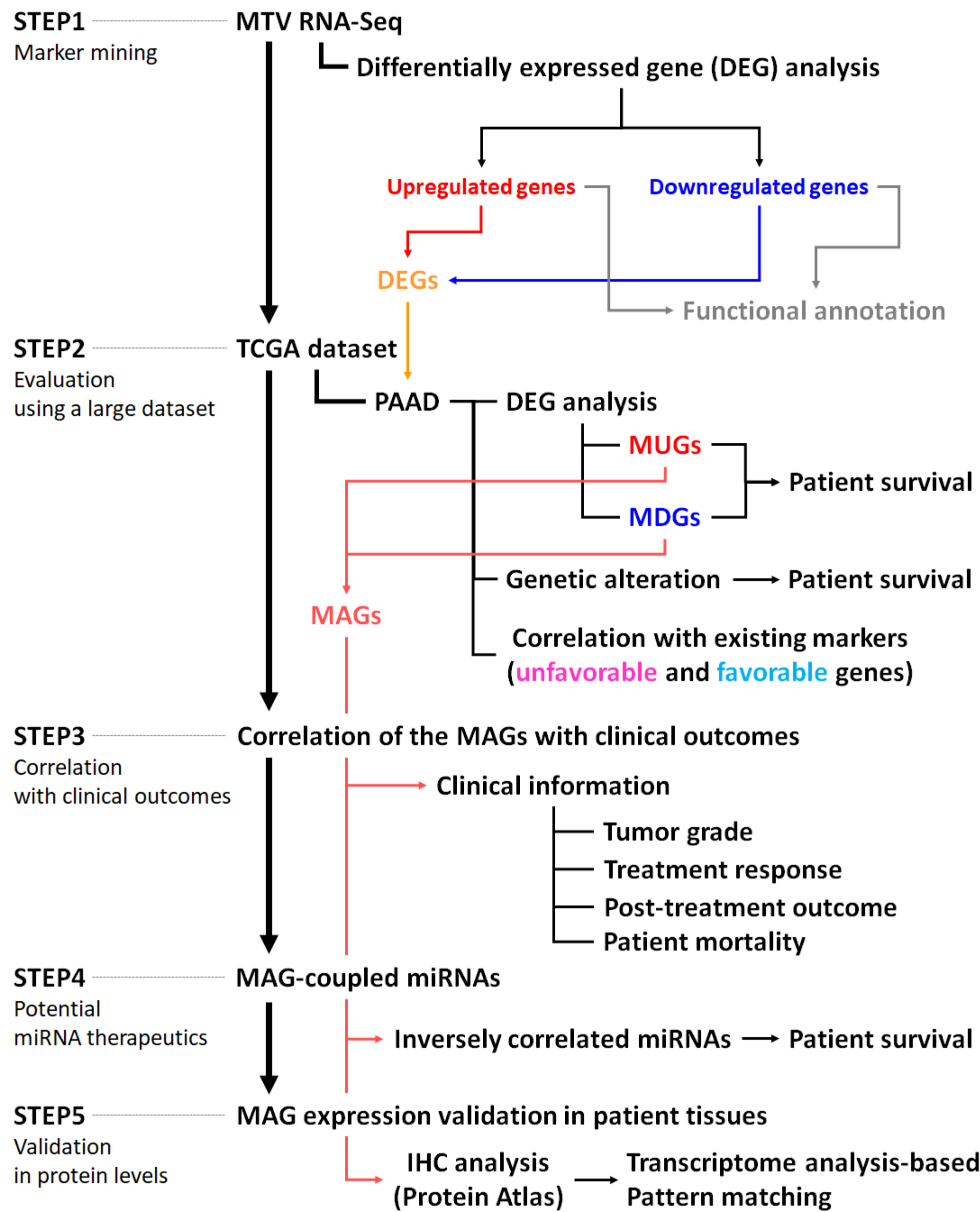

Supplement: Supplementary file 1 — Additional file 1: Figure S1. Data analysis workflow. Step 1. MTV RNA-Seq analysis. The initial stage involved conducting MTV RNA-Seq analysis to examine the differentially expressed genes (DEGs) in the MTV-high patient group compared to the MTV-low patient group. Subsequently, these DEGs were further studied to identify and analyze potential biological processes associated with them. Step 2. Application of MTV RNA-Seq analysis to the TCGA-PAAD dataset. The DEGs identified in Step 1 were further analyzed within the TCGA-PAAD dataset, which does not contain MTV information, to assess whether these DEGs could serve as reliable markers. The DEGs were designated as MTV-associated genes (MAGs) consisting of MTV-upregulated genes (MUGs) and MTV-downregulated genes (MDGs). Step 3. Correlation analysis between the DEGs and clinical outcomes. Using the clinical information of TCGA-PAAD, the correlation between MAGs and clinical outcomes was analyzed. Step 4. Potential miRNA therapeutics. MAG-coupled miRNAs' expression and patient survival were analyzed to find the potential miRNA therapeutics for pancreatic cancer. Step 5. Validation in protein levels. MAG expression was compared between pancreatic normal and cancer tissues and confirmed whether it matched the transcriptome analysis. [file 12967_2024_5181_MOESM1_ESM.pdf]

# Supplementary Figure S2.

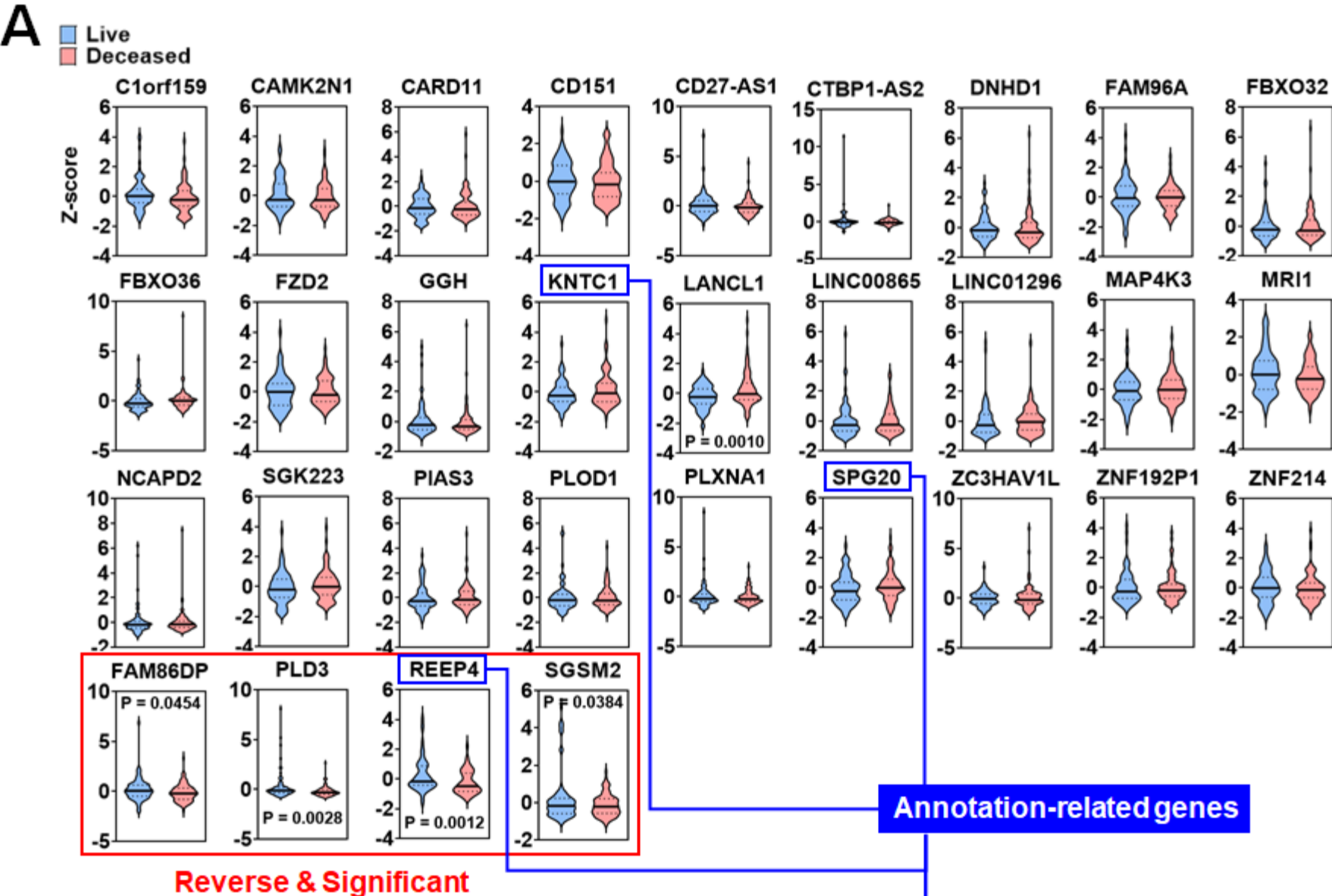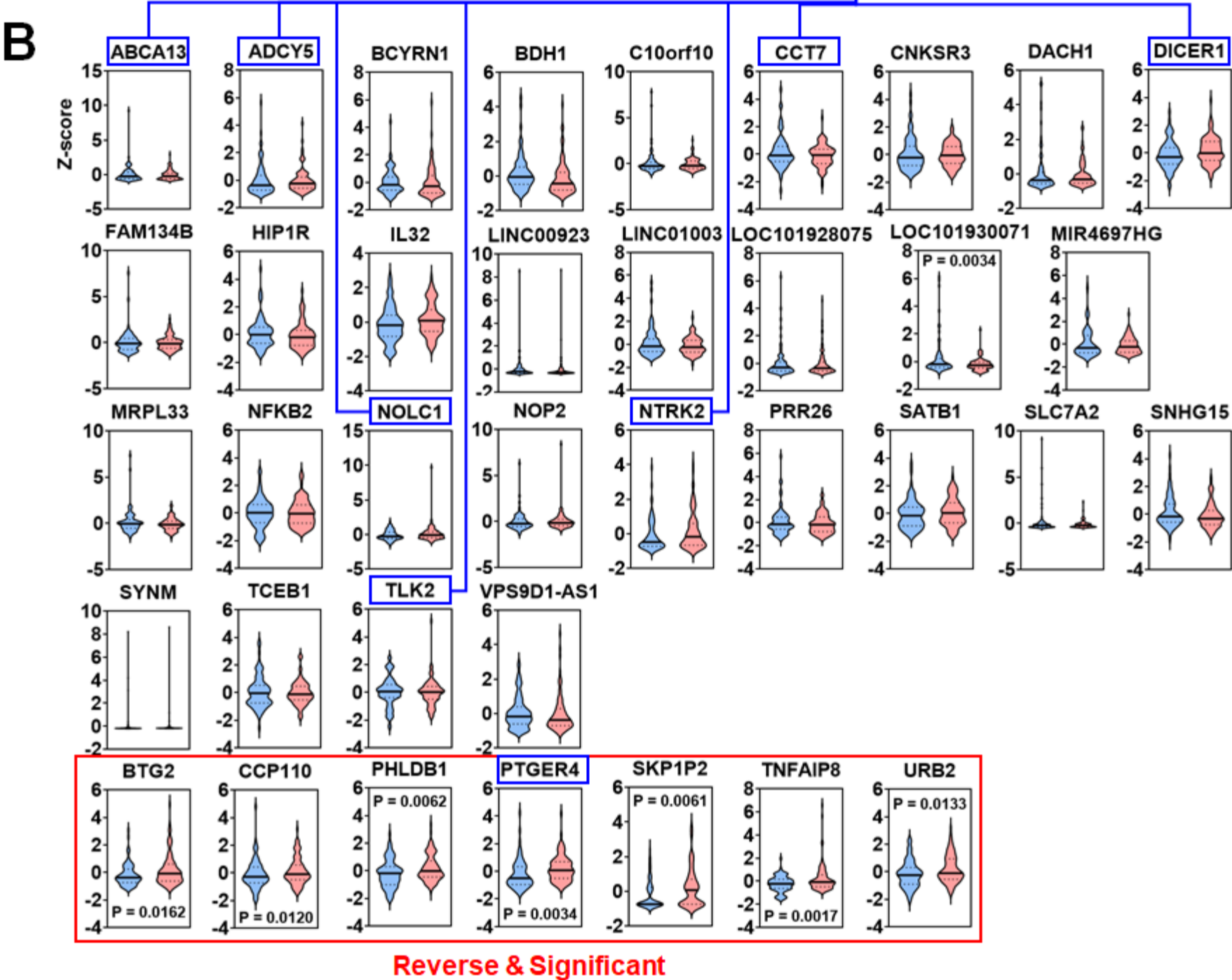

Supplement: Supplementary file 2 — Additional file 2: Figure S2. Expression graphs of MAGs that did not match the MTV RNA-Seq analysis. Expression graphs of A) MUGs and B) UDGs between live and deceased groups. Red-boxed genes are included in the enriched annotation. [file 12967_2024_5181_MOESM2_ESM.pdf]

Supplementary Figure S3.

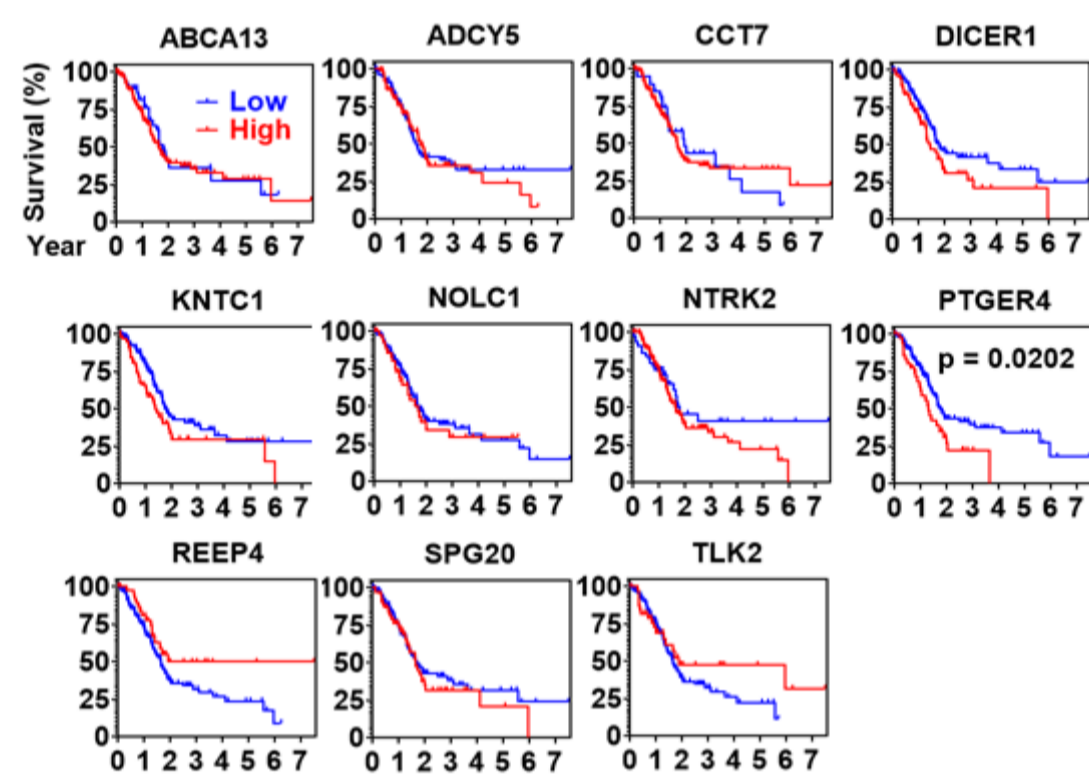

Supplement: Supplementary file 3 — Additional file 3: Figure S3. The survival comparison of patient groups with low and high expression of the genes involved in annotation but not MAGs. [file 12967_2024_5181_MOESM3_ESM.pdf]

Supplementary Figure S4.

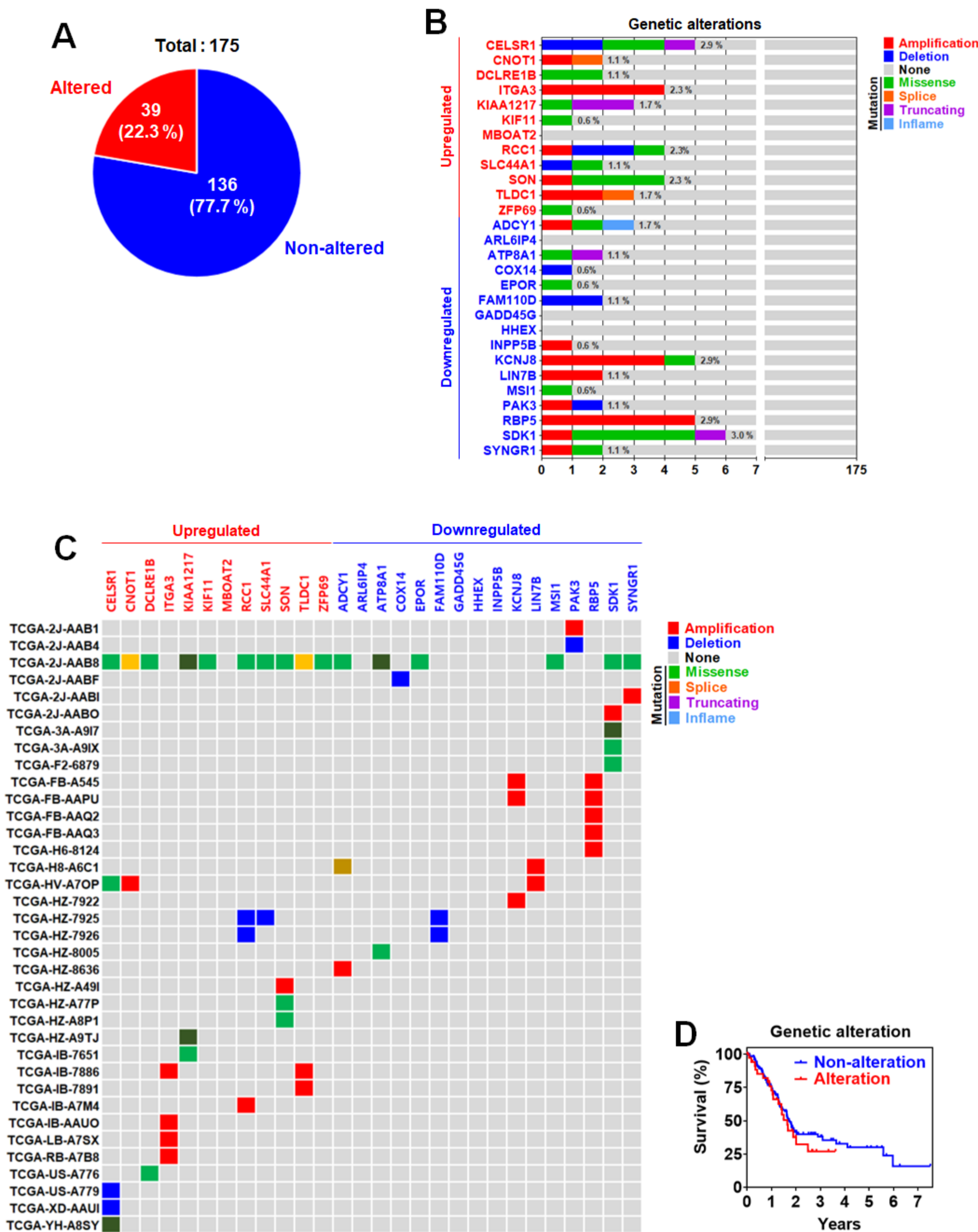

Supplement: Supplementary file 4 — Additional file 4: Figure S4. The genetic alteration of MAGs and its association with patient survival. A) Venn diagram presentation of the number of patients with or without MAG-related genetic alteration in TCGA-PAAD. B) The frequency of MAG-related genetic alterations in TCGA-PAAD. C) The distribution of MAG-related genetic alterations in patients. D) Survival comparison between the patient groups with or without MAG-related genetic alteration. [file 12967_2024_5181_MOESM4_ESM.pdf]

Supplementary Figure S5.

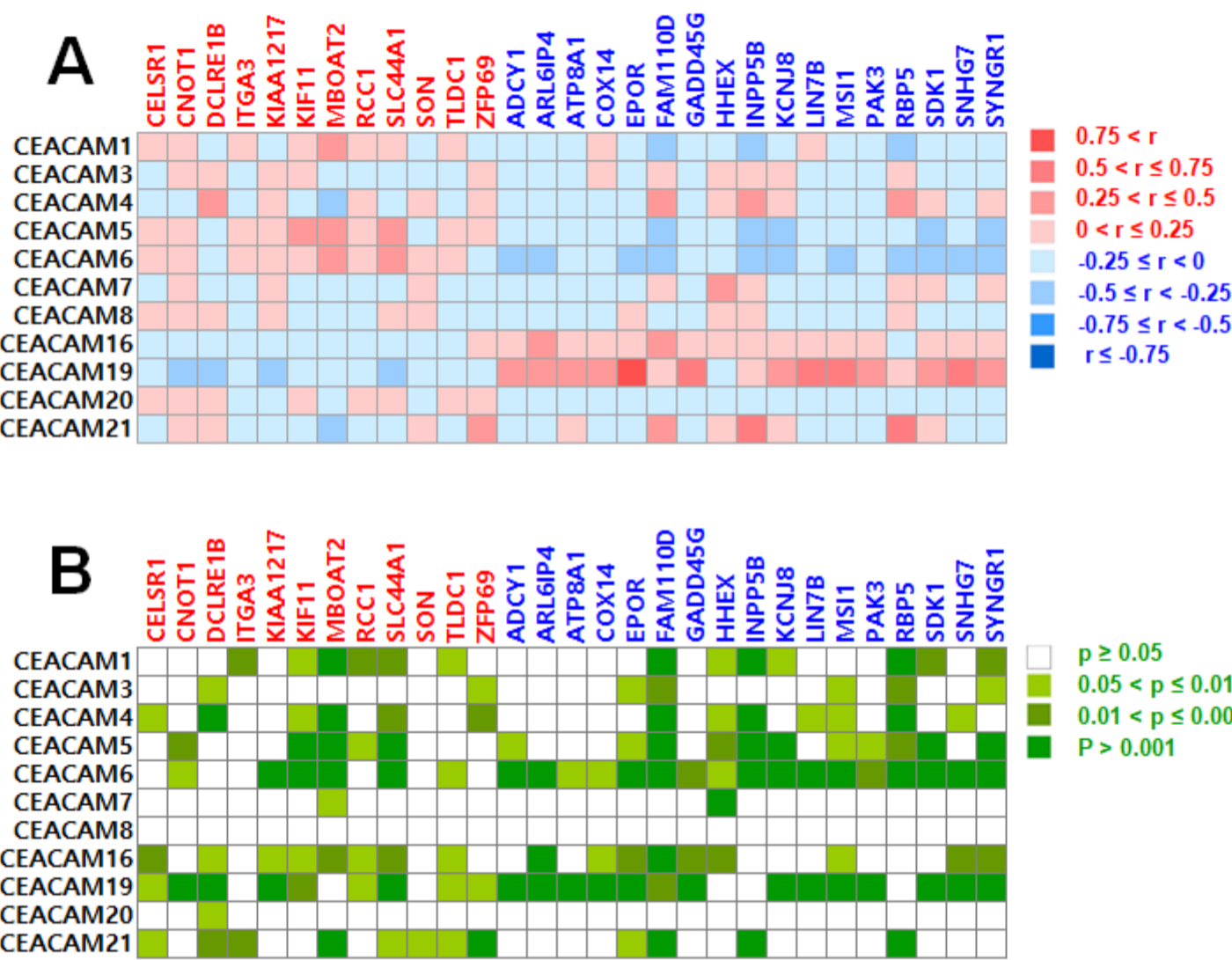

Supplement: Supplementary file 5 — Additional file 5: Figure S5. Correlation heatmap between MAGs and CEACAMs. The heatmap presentation of A) correlation coefficients (r) and B) p-values between MAGs and CEACAMs. [file 12967_2024_5181_MOESM5_ESM.pdf]

Supplementary Figure S6.

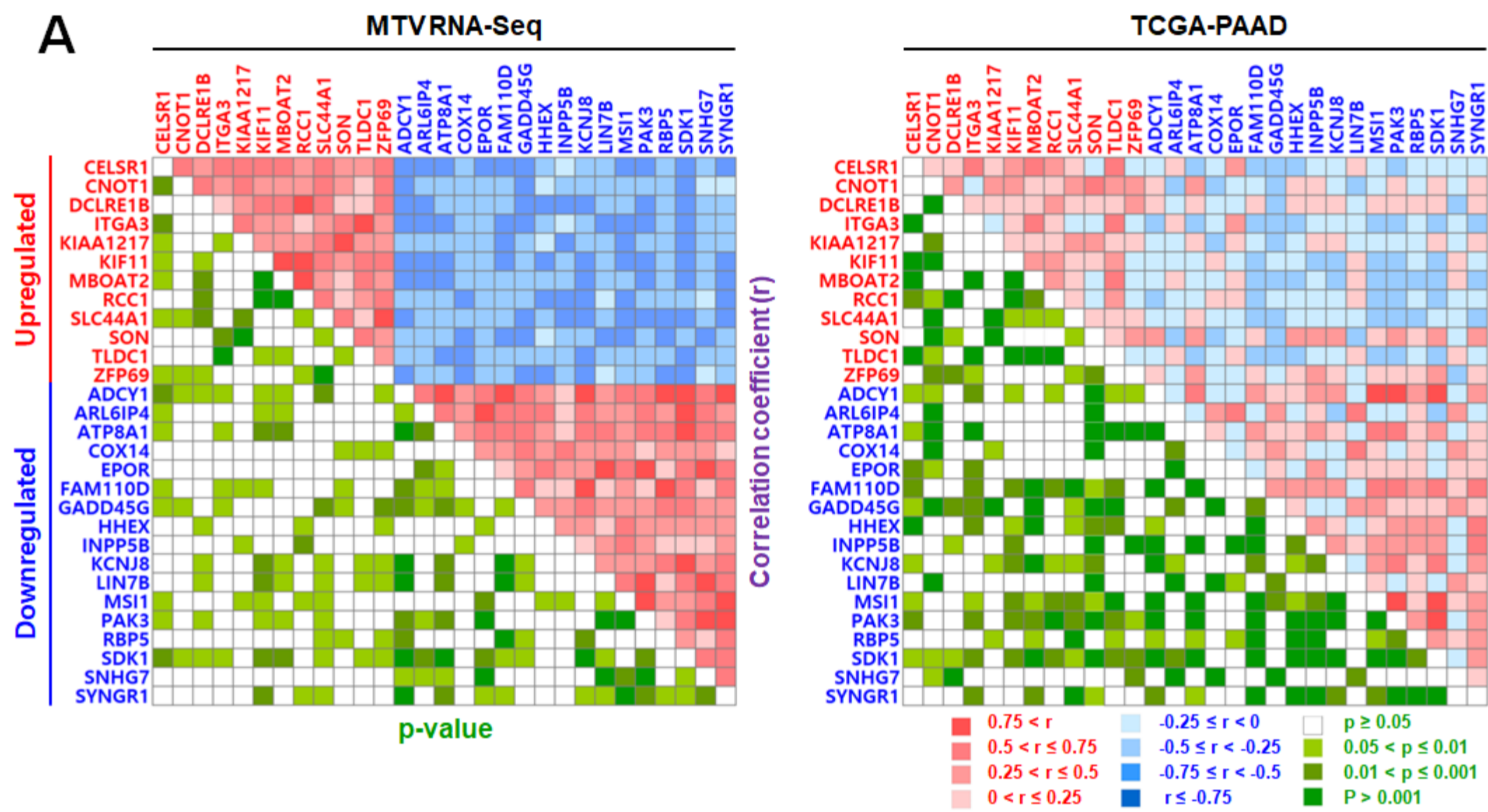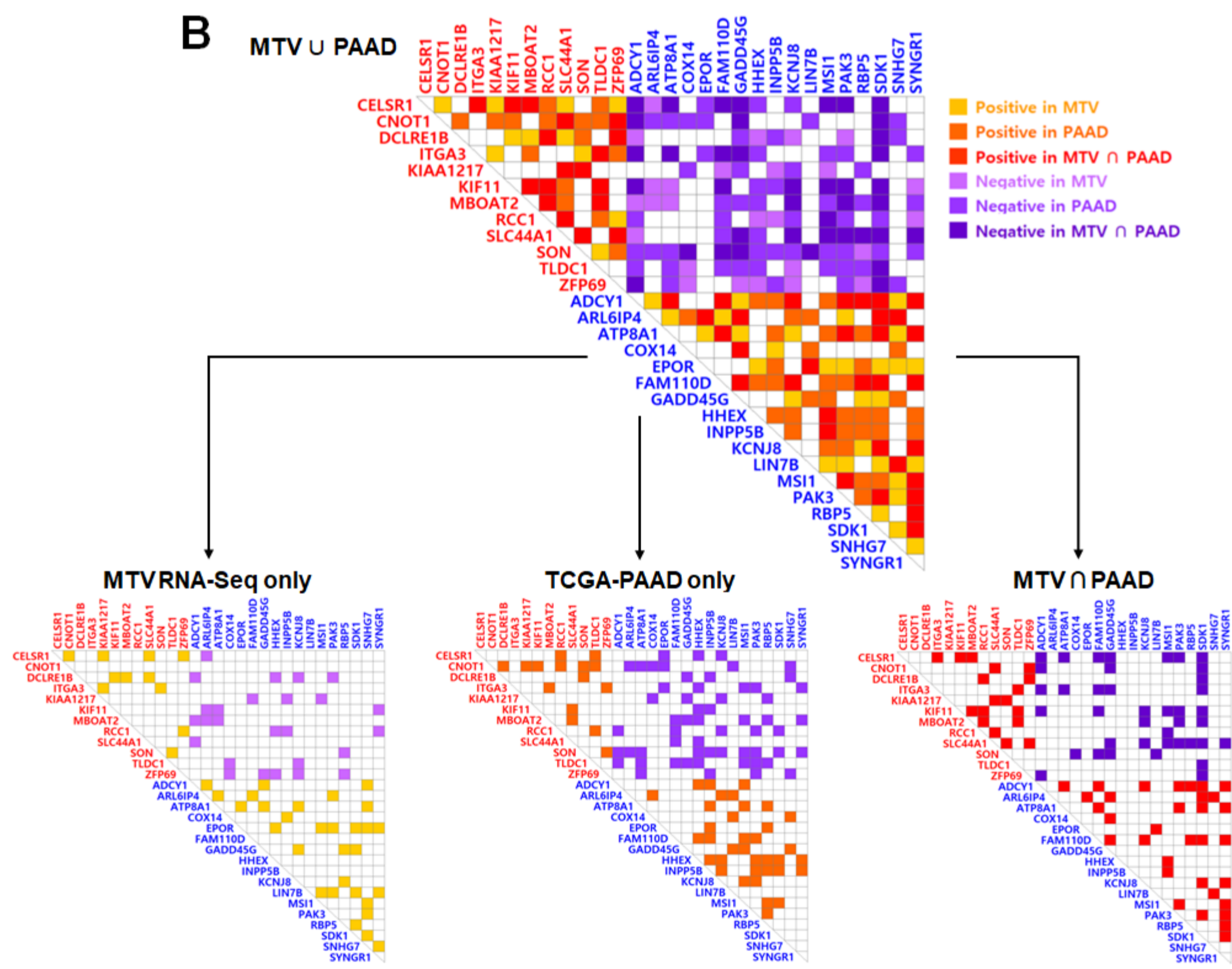

Supplement: Supplementary file 6 — Additional file 6: Figure S6. Correlation analysis of MAGs between MTV RNA-Seq and TCGA-PAAD dataset. A) The correlation matrix analysis of MAGs in MTV RNA-Seq and TCGA-PAAD. B) Statistically significant correlation in MTV RNA-Seq and TCGA-PAAD. The correlation analyses were presented in four categories, (1) MTV ∪ PAAD, (2) MTV RNA-Seq only, (3) TCGA-PAAD only, and (4) MTV ∩ PAAD. [file 12967_2024_5181_MOESM6_ESM.pdf]

Supplementary Figure S7.

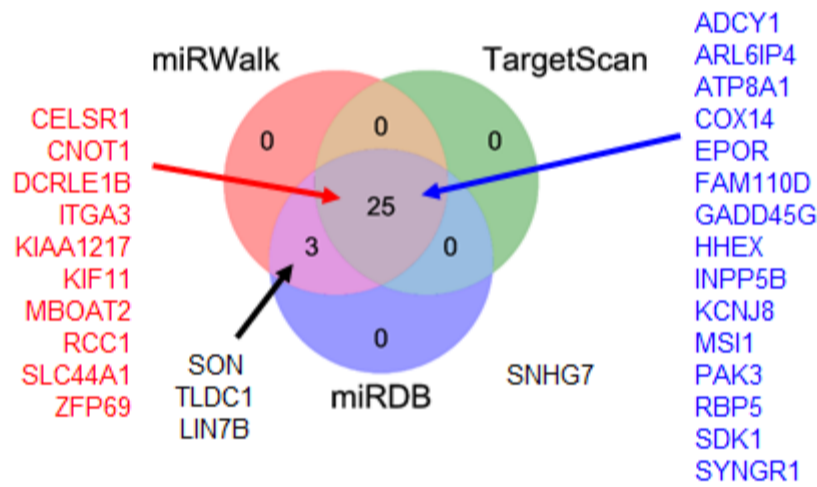

Supplement: Supplementary file 7 — Additional file 7: Figure S7. Venn diagram presentation of the databases analyzed for identifying the miRNAs targeting MAGs. 25 MAGs were commonly involved in all three databases. 3 genes were included in only two databases (miRWalk and miRDB). SNHG7 was not found in all three databases. [file 12967_2024_5181_MOESM7_ESM.pdf]

Supplementary Figure S8.

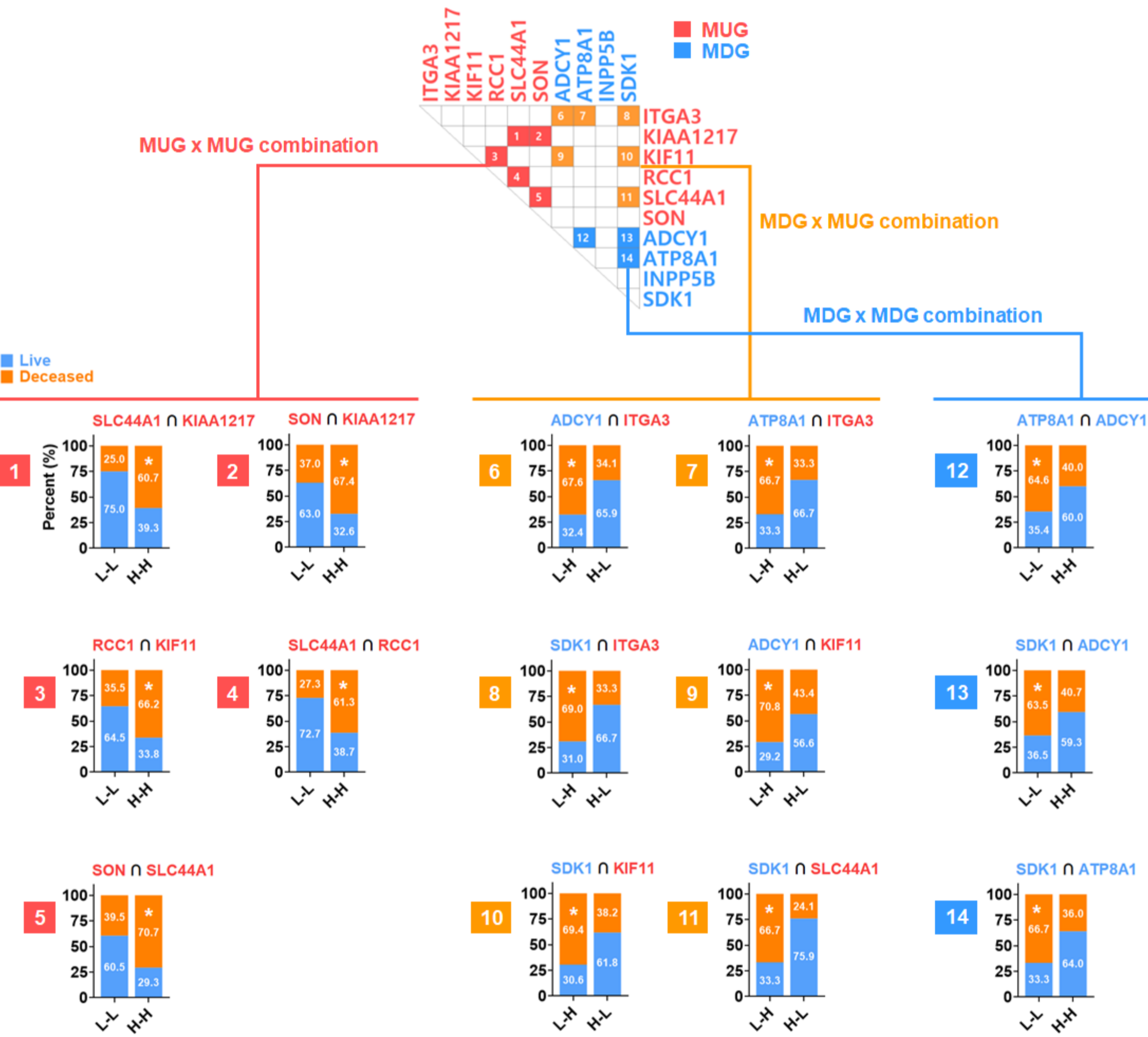

Supplement: Supplementary file 8 — Additional file 8: Figure S8. MAG combination-predicted mortality rates in stages I and II patients in the TCGA-PAAD dataset. The mortality rates were calculated in the patient group showing high expression of two MUGs for the MUG x MUG combination, low expression of an MDG and high expression of a MUG for the MDG x MUG combination, and low expression of two MDGs for the MDG x MDG combination. The numbers and colors of the half matrix match those of the bar graphs. Abbreviation: L-L: a combination of two low-expression MAGs; H-H: a combination of two high-expression MAGs; L-H: a combination of a low-expression MAG and a high-expression MAG; and H-L: a combination of a high-expression MAG and a low-expression MAG. White asterisks indicate the mortality rates calculated by MAG combinations. [file 12967_2024_5181_MOESM8_ESM.pdf]
